# Acute Phase Protein Orosomucoid (Alpha-1-Acid Glycoprotein) Predicts Delayed Cerebral Ischemia and 3-Month Unfavorable Outcome after Aneurysmal Subarachnoid Hemorrhage

**DOI:** 10.3390/ijms242015267

**Published:** 2023-10-17

**Authors:** Laszlo Zavori, Reka Varnai, Tihamer Molnar, Balazs Szirmay, Nelli Farkas, Attila Schwarcz, Peter Csecsei

**Affiliations:** 1 Emergency Department, Saudi German Hospital, Dubai 391093, United Arab Emirates; zavori.laszlo@gmail.com; 2 Doctoral School, University of Pecs, 7624 Pecs, Hungary; 3 Department of Primary Health Care, Medical School, University of Pecs, 7624 Pecs, Hungary; varnai.reka@pte.hu; 4 Department of Anaesthesiology and Intensive Care, Medical School, University of Pecs, 7624 Pecs, Hungary; tihamermolnar@yahoo.com; 5 Department of Laboratory Medicine, Medical School, University of Pecs, 7624 Pecs, Hungary; 6 Institute of Bioanalysis, Medical School, University of Pecs, 7624 Pecs, Hungary; nelli.farkas@aok.pte.hu; 7 Department of Neurosurgery, Medical School, University of Pecs, 7624 Pecs, Hungary; schwarcz.attila@pte.hu (A.S.); csecsei.peter@pte.hu (P.C.)

**Keywords:** aneurysmal subarachnoid hemorrhage, orosomucoid, alpha-1-acid glycoprotein, delayed cerebral ischemia, outcome

## Abstract

The pathophysiology and consequences of early brain injury (EBI) after aneurysmal subarachnoid hemorrhage (aSAH) remain incompletely understood. This study aims to investigate the role of orosomucoid (ORM) in aSAH, its potential as a marker for assessing the extent of EBI-induced damage, and its correlation with delayed cerebral ischemia (DCI) and functional recovery over a 3-month period. We collected serum specimens 72 h post-aSAH to measure ORM levels. The study included 151 aSAH patients and 105 healthy subjects. The serum ORM levels within the patient cohort significantly exceeded those in the control group (*p* < 0.001). The ORM value showed significant correlation with the admission WFNS (*p* < 0.0001) and mFS scores (*p* < 0.05). Substantially elevated serum ORM levels at 72 h post-aSAH were detected among patients experiencing DCI, as well as those with poor functional outcomes after 3 months (*p* = 0.009 and *p* < 0.001). Binary logistic regression analyses revealed that serum ORM at 72 h post-SAH was independently associated with DCI and 3-month functional outcome after adjusting for confounders. The early stage events of aSAH influence the level of ORM. ORM serves as a marker for assessing the extent of damage during EBI and is linked to the occurrence of DCI as well as unfavorable long-term functional outcomes.

## 1. Introduction

Aneurysmal subarachnoid hemorrhage (SAH) is a devastating disease which is associated with a high risk of death or severe disability [1]. Early brain injury (EBI)—the set of harmful pathophysiological processes occurring within 72 h after aneurysm rupture—has become the focus of SAH research in recent years [2]. During this early period, many patients develop secondary brain damage, which can be attributed to several pathological mechanisms, such as microcirculatory disturbances, breakdown of the blood–brain barrier (BBB), neuroinflammation, oxidative cascades, brain edema, and neuronal death [3]. Following a subarachnoid hemorrhage (SAH), the brain displays a markedly pro-inflammatory phenotype, during which there is a significant increase in the expression of the cytokines IL-6, IL-10, TNF-α, and IL-1β [4]. α-1-acid glycoprotein, also referred to as orosomucoid, is an acute-phase protein primarily synthesized in the liver. However, it is also synthesized in extrahepatic sites such as granulocytes or myocardial cells [5,6]. In response to acute inflammation, its concentrations undergo a substantial rise [7], thereby modulating the immune system as part of the acute-phase reaction [5,7]. ORM gene expression is primarily regulated by a combination of regulatory mediators such as glucocorticoids, interleukin (IL)-1, TNF-α, and IL-6 [8]. Delayed cerebral ischemia (DCI) is a serious complication of aSAH that increases the risk of morbidity and mortality [9]. EBI is a known precursor of DCI [2]; however, its precise pathogenesis remains incompletely understood. Our hypothesis assumes that the processes taking place during early brain injury (EBI) influence the level of ORM, which, as an indicator of the inflammatory processes taking place during EBI, shows a correlation with the most serious complication of aSAH and the long-term functional outcome. The primary objective of our study is to establish a correlation between three key elements: (i) the long-term (3-month) functional outcome, serving as the primary endpoint, (ii) the incidence of delayed cerebral ischemia (DCI) as the secondary endpoint, and (iii) inflammatory markers measured upon admission and the serum concentration of orosomucoid in patients with subarachnoid hemorrhage caused by aneurysm rupture.

## 2. Results

### 2.1. Cohort Characteristics

**Figure 1:**
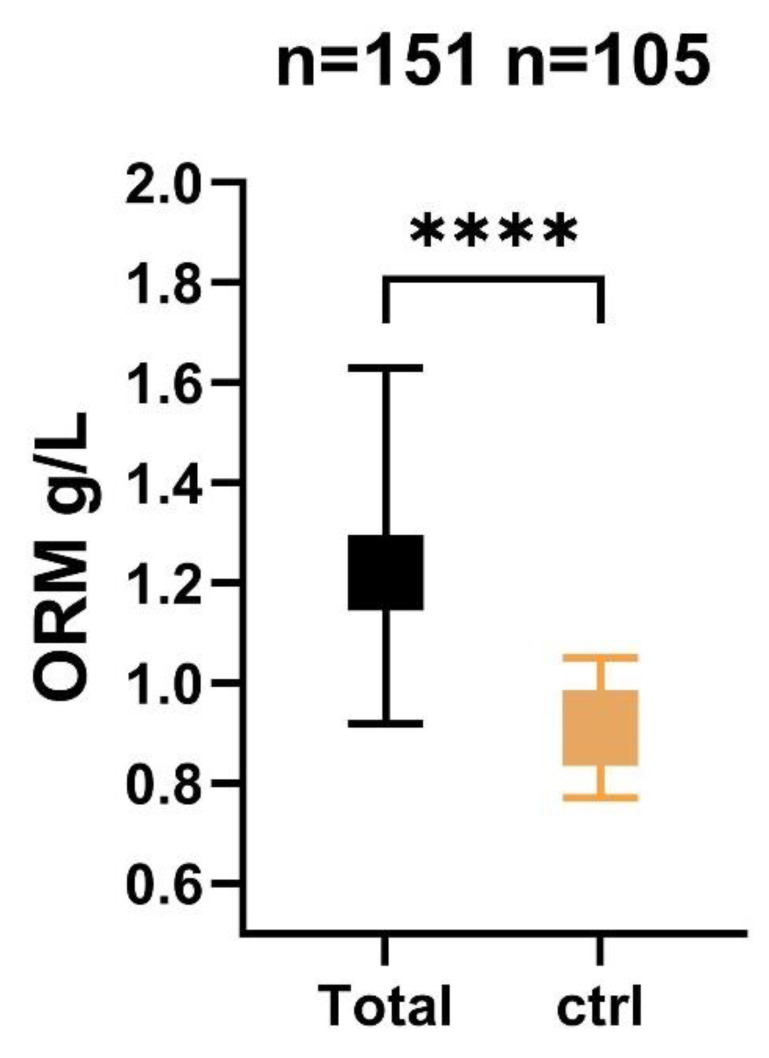
Level of serum ORM measured 72 h post-SAH in patients and control cases. ORM, orosomucoid; ctrl, control; n, number; SAH, subarchnoid hemorrhage; **** *p* < 0.0001.

The final analysis included a total of 151 patients who were enrolled in the study between December 2020 and January 2023. The mean age of the cohort was 55 ± 12 years, and 69.5% were female. Admission serum median CRP levels were 14 mg/dL [IQR: 4–48]. Baseline characteristics stratified by 3-month functional outcome are summarized in Table 1. A total of 100% of the aneurysms were secured by coiling. Almost half of the patients had a history of arterial hypertension (47%) and 11% had a history of NIDDM. A total of 24.5% of the patients were hospitalized in a poor grade (WFNS IV–V) condition. The serum ORM (g/L) level of the patient’s cohort was 1.22 (IQR 0.94–1.60), while the median ORM level of the 105 healthy control subjects was 0.91 (0.77–1.05), *p* < 0.001, Figure 1. The median age of the control cases was 58 years (interquartile range (IQR) 49–66), and 58.4% were women. ROC curve analysis was performed and demonstrated that serum ORM levels were able to distinguish patients with aSAH from the control cases, with an area under the curve of 0.771 (95% CI 0.702 to 0.840), *p* < 0.001.

### 2.2. Association of Serum ORM Level with Admission Severity Scores

**Figure 2:**
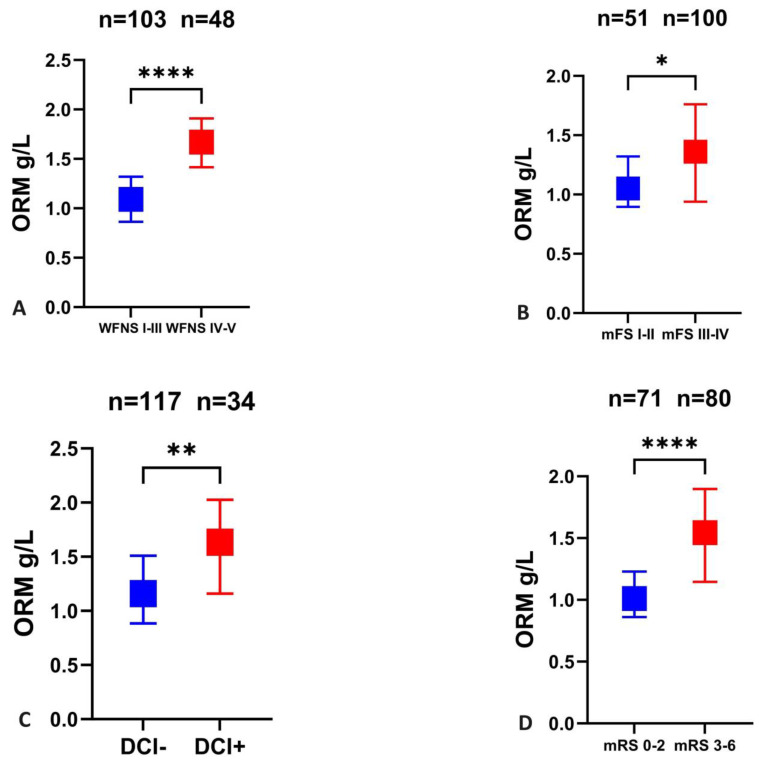
Serum levels of ORM in different severity and outcome groups. (**A**) Serum levels of ORM in SAH patients with good (WFNS I–III) and poor (WFNS IV–V) grade on admission. (**B**) ORM levels in low (mFS I–II) and high (mFS III–IV) radiological grade of SAH. Comparisons between patients with and without DCI (**C**) as well as between favorable (mRS 0–2) and unfavorable (mRS 3–6)-3 month outcome (**D**) after aneurysmal subarachnoid hemorrhage in terms of serum ORM concentrations. ORM, orosomucoid; mFS, modified Fisher score; WFNS, World Federation of Neurological Societies score; n, number; DCI, delayed cerebral ischemia. * *p* < 0.05, ** *p* < 0.01,**** *p* < 0.0001.

Serum ORM measured at 72 after ictus was significantly higher in patients with a poor grade (WFNS IV–V) condition on admission compared to those with a good clinical state (WFNS I–III) on admission (1.7 [IQR 1.5–1.9] vs. 1.1 [0.9–1.3], *p* < 0.001), Figure 2A. A similar but weaker correlation can be observed between the serum ORM level and admission mFisher-score (mFS I + II: 1 [0.9–1.3] vs. mFS III + IV: 1.4 [0.9–1.8], *p* = 0.021, Figure 2B.

### 2.3. Serum ORM Levels in Relation to DCI and 3-Month Outcome

Significantly higher levels of serum ORM at 72 h post-SAH were detected in patients with the presence of DCI and with poor functional outcome at 3 months (*p*-value = 0.009 and <0.001, respectively, Figure 2C,D). Binary logistic regression analyses revealed that serum ORM at 72 h post-SAH was independently associated with DCI after adjusting for age, sex, mFS, and WFNS. In addition, ORM was independently associated with poor mRS at 3 months after adjusting for age, sex (Model a), and mFS (Model b) and WFNS grade (Model c), Table 2. The area under the curve of serum ORM for discriminating between patients with and without DCI was 0.713 (95% CI 0.562 to 0.864), Table 3. Subsequent ROC curve analysis demonstrated that serum orosomucoid (ORM) levels assessed at the 72 h mark following the onset of symptoms were able to distinguish patients with a favorable 3-month outcome (mRS 0–2) from those with an unfavorable 3-month outcome (mRS 3–6), with an area under the curve of 0.793 (95% CI 0.694 to 0.891, *p* < 0.001), as shown in Table 3.

### 2.4. Variables Associated with Higher Serum ORM Level

The results of univariate binary logistic regression analysis indicated a strong association between several characteristics and elevated orosomucoid (ORM) concentrations subsequent to aneurysmal subarachnoid hemorrhage, Table 4.

## 3. Discussion

In this study, we investigated how serum ORM levels measured 72 h after stroke correlate with the 3-month functional outcome, the occurrence of delayed cerebral ischemia (DCI) during hospitalization, and their connection with admission inflammatory markers. Regarding the primary endpoint, we found that serum orosomucoid levels were significantly higher in the group with an unfavorable 3-month outcome compared to the group with a favorable outcome. Furthermore, elevated serum orosomucoid emerged as an independent predictor of a poor 3-month outcome in patients with aSAH. In a previous small-scale study, elevated levels of orosomucoid were also observed in the cerebrospinal fluid (CSF) of patients who suffered from hemorrhagic stroke [10]. Global cerebral edema and intracranial pressure (ICP) crisis were significantly associated with mortality after adjustment of admission predictors of mortality in a large cohort of aSAH patients [11]. 

Microvascular endothelial cells normally produce orosomucoid, which is essential for capillary barrier [12]. Increased capillary permeability accompanied by edema may arise from compromised synthesis of orosomucoid and other vital constituents of the glycocalyx, a consequence potentially attributed to endothelial dysfunction [12]. The addition of orosomucoid to the resuscitation fluid increased circulating blood volume, reduced edema formation, and neutrophil accumulation following trauma and hemorrhagic shock [13]. Orosomucoid possesses the capacity to regulate the permeability of the blood–brain barrier (BBB) to charged molecules by introducing negative charge to the matrix components of the BBB. This mechanism aids in upholding the BBB’s low permeability state during both physiological and pathological conditions [14]. The cerebral edema that occurs after SAH, which is a predictor of poor outcome, likely indirectly increases orosomucoid production to improve the compromised blood–brain barrier function. EBI significantly contributes to a poor outcome after subarachnoid hemorrhage [2,3]. ORM was reported to activate the TLR4/CD14 signaling pathway [15], which is a known mechanism of EBI after aSAH [3]. Apart from orosomucoid, hemorrhagic blood products activate TLR-4, triggering de novo synthesis of inflammatory cytokines and subsequently inducing inflammation in the central nervous system. This cascade ultimately contributes to an unfavorable clinical outcome [16]. Other human studies have demonstrated that elevated TLR-4 levels in peripheral macrophages are associated with poor functional recovery after aSAH [17]. The high level of ORM in patients with a poor 3-month outcome may be an indicator of BBB damage and enhances the negative effects of neuroinflammation through the TLR4 pathway during EBI, contributing to an unfavorable outcome in aSAH patients. 

Another important result of our study is that the 72 h ORM level is an independent predictor of later DCI. It is known that vascular dysfunction, microthrombosis, neuroinflammation, and oxidative stress are the pathophysiological factors in the development of DCI [18]. ORM directly contributes to platelet activation, which in turn might have an impact on physiological hemostasis and/or pathological thrombosis [19]. Nevertheless, orosomucoid in high concentrations has also been shown to inhibit platelet aggregation [20]. This concentration-dependent effect on hemostasis might play a role in the thrombotic processes of DCI. The involvement of glia in aSAH pathology was established when it was demonstrated that microglia-depleted mice have reduced vasospasm and neuronal apoptosis [16]. The same study showed that neuronal apoptosis and vasospasm are diminished in TLR4 knockout mice early after aSAH [16]. This early period coincides with the time of EBI. ORM is a novel mediator of astrocyte–microglia interaction in the CNS [21]. Reactive astrocytes within the inflamed brain demonstrate pronounced ORM expression, and heightened ORM protein levels are associated with a dampened microglia-mediated neuroinflammatory response [21]. Free heme is a TLR4 activator [22] and, thereby, enhances neuronal apoptosis via this route. In murine models, ORM synthesized by endothelial cells has been linked to anti-apoptotic effects during ischemic-perfusion injury [23]. Moreover, ORM modulates injury-induced angiogenesis in a context-dependent and time-sensitive manner [24]. ORM’s influence extends to the enhancement of CD163 expression—a pivotal element for efficient extracellular hemoglobin (Hb) clearance. Furthermore, AGP also demonstrates anti-inflammatory properties in mitigating oxidative stress arising from hemolysis-induced mechanisms [15]. On one hand, orosomucoid (ORM) contributes to pathways that lead to adverse outcomes, such as escalated TLR4 expression and proapoptotic effects. Conversely, ORM also possesses the ability to trigger counteractive processes. It is plausible that the prevailing effect is contingent on the localized concentration of ORM. This dual stimulatory and inhibitory combined function is already known in relation to angiogenesis [24]. To confirm the dose-dependent effects, further studies at the cellular and tissue level are needed to clearly clarify the significance of ORM in signaling the pathophysiological changes occurred during EBI. From our study, it can be inferred that the strongest predictive factor of serum ORM levels is the severity of symptoms at admission; other demographic factors (gender, age, smoking) do not influence the serum level. This also confirms that ORM can be a promising indicator of pathophysiological damage occurred during EBI.

Finally, we found that serum ORM showed a strong positive correlation not only with the WFNS and mFS scores, but also with admission CRP and neutrophil count. A strong degree of positive correlation is observed within levels of CRP and ORM in several inflammatory conditions such as experimental periodontitis [25], bacterial infection in neonates [26], and psoriasis [27]. While a clear positive correlation exists between admission CRP, neutrophil count, and the elevated serum ORM value at 72 h, it is important to acknowledge that the varying sampling times and the absence of knowledge regarding the kinetics of inflammatory parameters and ORM prevent us from making definitive conclusions regarding the precise pathophysiological significance of the observed correlation and its relevance in EBI processes. This limitation is one of several within our study. Additional limitations that should be noted: We performed a solitary serum measurement, thereby lacking data on fluctuations in serum ORM levels over time. Our investigation did not encompass other processes contributing to the pathogenesis of early brain injury (EBI). Additionally, our analysis solely relied on inflammatory markers available at the time of admission, which restricts the comprehensive contextual understanding of our findings.

## 4. Materials and Methods

### 4.1. Study Population

This was a prospective observational study from a stroke treatment center in Pecs, Hungary. All patients aged ≥ 18 years, who were newly diagnosed with aneurysmal subarachnoid hemorrhage (aSAH) and admitted to our hospital between December 2020 and January 2023, were included in this study. Institutional review board approval was obtained previously (IV/8468-1/2021/EKU), and written informed consent was obtained from each patient or their legal representative. Inclusion criteria were: age > 18 years, spontaneous aneurysmal SAH diagnosed by computed tomography (CT) within 24 h of ictus, aneurysm detected on digital subtraction angiography (DSA). Exclusion criteria were: traumatic SAH, pregnancy, hospital admission later than 24 h after ictus, no aneurysm treatment, bleeding from arteriovenous malformation, absence of a signed consent form, underlying SARS-CoV-2 infection, systemic diseases (chronic neurological disease, tumors, liver and/or renal insufficiency, and chronic lung disease). All patients received treatment in accordance with the previously detailed clinical protocol [28]. Concurrently, a healthy control group consisting of 105 individuals, matched for age and gender, was established.

### 4.2. Clinical and Outcome Data Definitions

Common underlying health conditions (such as hypertension and diabetes), smoking history, and treatment-related complications (such as macrovascular vasospasm and delayed cerebral ischemia) were identified. Admission laboratory measurements and the location of the aneurysm were obtained from hospital records. The severity of patients’ clinical and radiological status upon admission was evaluated using the WFNS and modified Fisher scoring systems. The low-grade group was defined as follows: WFNS I–III on admission clinical examination or mFisher score I–II at first CT scan. The high-grade group was defined as WFNS IV–V or mFisher score III–IV on admission evaluation. Macrovascular vasospasm (MVS) was defined as narrowing of the arterial vessel lumen on MRI or cerebral angiography. Presence of MVS was assessed by visual inspection of cerebral angiography or the source images of cerebral angiography. Validation of the diagnosis required confirmation by an impartial observer, specifically, a neuroradiologist. The definition of clinical deterioration caused by delayed cerebral ischemia (DCI) is the occurrence of focal neurological impairment (such as hemiparesis, aphasia, apraxia, hemianopia, or neglect), or a decrease of at least 2 points on the Glasgow Coma Scale, which is not apparent immediately after aneurysm occlusion and cannot be attributed to other causes. The definition of cerebral infarction caused by DCI is: The presence of cerebral infarction on CT or MR scan of the brain within 6 weeks after SAH, or on the latest CT or MR scan made before death within 6 weeks, or proven at autopsy, not present on the CT or MR scan between 24 and 48 h after early aneurysm occlusion, and not attributable to other causes such as surgical clipping or endovascular treatment. Hypodensities on CT imaging resulting from ventricular catheter or intraparenchymal hematoma should not be regarded as cerebral infarctions from DCI [9]. DCI was diagnosed only after rigorous exclusion of other possible causes, and it was judged by the consensus of at least two neurointensivists. All cerebral CT scans on admission were graded for Subarachnoid Hemorrhage Early Brain Edema Score (SEBES) by independent raters blinded to clinical data [29]. SEBES is a semiquantitative score that measures cerebral edema on cranial CT on the basis of two CT levels and can be used to predict outcome after SAH. The definition criteria of systemic infection were as follows: symptoms of infection with fever, elevated C-reactive protein and/or procalcitonin, and a positive diagnostic test such as chest X-ray or urine test. The 3-month mRS was obtained via a personal visit by a trained research personnel. Unfavorable outcome at 3 months after discharge was defined as an mRS of 3–6 [30]. Subjects were treated according to standard guidelines.

### 4.3. Sample Collection and Processing Protocol

Serum blood samples were drawn into Vacutainer^®^—tubes from patients at 72 h after ictus to determine concentrations of ORM. The samples were centrifuged within 10 min at 3500 rpm for 15 min. The supernatant was immediately stored in aliquot at −80 °C until further processing. Serum ORM was measured by automated immune turbidimetric method using commercially available kits (Tina-quant α1-Acid Glycoprotein Gen.2, Roche Diagnostics GmbH, Basel, Switzerland) from the Department of Laboratory, University of Pecs, Hungary. We used an age-matched healthy population as a control group.

### 4.4. Statistical Analysis

The normal distribution was assessed using the Shapiro–Wilk test. Categorical variables are presented as number and percentages, while continuous variables are presented as medians and IQRs. Two-group comparisons were performed using the Mann–Whitney U test (continuous variables) or the χ^2^ test (categorical variables). Spearman’s rank correlation was analyzed by bivariate correlations. The relationship between ORM and the two endpoints (poor outcome (defined as a mRS score of 3–6 points at 3 months) and occurrence of DCI) was also investigated using regression analysis. Crude and adjusted results are presented as OR and 95% CI, respectively. During the multivariate regression analysis, we used the variance inflation factor (VIF) to test for multicollinearity, where a VIF above 4 was considered indicative of potential multicollinearity. A receiver operating characteristic (ROC) curve was made for ORM predicting outcome and DCI, along with estimation of area under the curve (AUC), sensitivity, and specificity of optimal cut-off value (calculated by Youden’s index). In line with current statistical consensus, an AUC of 0.8–0.9 is considered very good, 0.7–0.8 is considered adequate, and <0.7 is considered poor in terms of accuracy of the test under consideration. A *p* value of <0.05 was considered significant. SPSS 19.0 (SPSS Inc., Chicago, IL, USA) and Graph Pad Prism 9 software (GraphPad Software, San Diego, CA, USA) was used for statistical analysis of data.

## 5. Conclusions

In the early stages of aSAH, ORM may play an important role as an indicator of EBI-induced damage. Further investigations are essential to gain a comprehensive understanding of its precise role and significance in this context.

## Figures and Tables

**Table 1:** Patients’ characteristics.

	Favorable (N = 71)	Unfavorable (N = 80)	*p*-Value
Age, years	52 ± 12	58 ± 12	0.002
Female	51 (72)	65 (81)	0.529
Hypertension	31 (44)	41 (51)	0.393
NIDDM	3 (4)	14 (17)	0.015
Smoking	14 (20)	14 (18)	0.754
mFisher score	3 (2–3)	3 (3–4)	0.001
WFNS	1 (1–2)	4 (2–5)	<0.001
SEBES	1 (1–2)	3 (1–4)	<0.001
Aneurysm location			
ICA	11 (15)	7 (9)	0.670
MCA	18 (25)	22 (28)	0.325
ACoA	25 (36)	26 (33)	0.410
PCom	6 (9)	6 (7)	0.980
ACA	3 (4)	8 (10)	0.088
VB	8 (11)	11 (13)	0.650
CRP (mg/L)	8.3 (3–17)	27 (9–73)	<0.001
NLR	4.2 (3–7)	6.4 (4–11)	0.002
WBC (G/L)	11.4 (8.7–13.4)	12.2 (10.4–15.7)	0.028
Neutrophile (G/L)	8 (6.5–10.8)	9.9 (7.5–12.9)	0.015
Lymphocyte (G/L)	1.7 (1.1–2.3)	1.4 (1.1–2)	0.093
Glucose (mmol/L)	7.2 (6.2–9.4)	8.1 (7.3–9.5)	0.145
Creatinine	60 (51–68)	61 (49–73)	0.654
Hydrocephalus	23 (32.4)	59 (73.8)	<0.001
Intraparenchymal hematoma	2 (3)	19 (24)	<0.001
Extraventricular drainage	12 (17)	57 (71)	<0.001
Decompressive craniotomy	0 (0)	19 (24)	<0.001
Mechanical ventilation	7 (10)	60 (75)	<0.001
infection	6 (10)	26 (32)	0.004
Macrovascular vasospasm	12 (17)	30 (37)	0.007
Delayed cerebral ischemia	4 (7)	30 (37)	<0.001

Values are expressed as numbers (% of total), mean ± SD or median (IQR). N, number; NIDDM, non-insulin dependent diabetes mellitus; NLR, neutrophil-lymphocyte ratio; WFNS score, World Federation of Neurosurgical Societies score; ICA, internal carotid artery; MCA, middle cerebral artery; ACoA, anterior communicating artery; PCom, posterior communication artery; ACA, anterior cerebral artery; VB, vertebrobasilar; CSF, cerebrospinal fluid; DCI, delayed cerebral ischemia; WBC, white blood cell count; SEBES, Subarachnoid Hemorrhage Early Brain Edema Score.

**Table 2:** Adjusted odds ratio of serum ORM for prediction of DCI and poor functional outcome at 3 months.

Variable	Multivariate		
	OR	95% CI	*p*-Value
DCI			
Model ^a^	1.227	1.068–1.410	0.004
Model ^b^	1.467	1.091–1.971	0.011
Model ^c^	1.574	1.095–2.260	0.014
Poor clinical outcome at 3 months			
Model ^a^	1.518	1.257–1.833	<0.001
Model ^b^	1.432	1.145–1.791	0.002
Model ^c^	1.308	1.016–1.684	0.037

DCI, delayed cerebral ischemia; OR, odds ratio; CI, confidence interval; mFS, modified Fisher score; WFNS, World Federation of Neurological Societies score; ^a^ Adjustments were made for age and sex; ^b^ Adjustments were made for age, sex, and mFS; ^c^ Adjustments were made for age, sex, mFS, and WFNS grade. Poor clinical outcome defined as modified Rankin score 3–6.

**Table 3:** ROC analysis for serum ORM level (g/L), as a continuous variable associated with DCI and poor functional outcome at 3 months.

	Cut-off Value	AUC	*p*-Value	95% CI	Sensitivity (%)	Specificity (%)	Power
DCI	1.270	0.713	0.009	0.562–0.864	68.8	60	AC
Poor functional outcome at 3 months	1.200	0.793	<0.001	0.694–0.891	72.5	68.3	AC

ROC, receiver operating characteristic; AUC, area under the curve; CI, confidential interval; DCI, delayed cerebral ischemia; ORM, orosomucoid; power, ability to diagnose patients with and without the disease or condition based on the test, AUC of 0.8–0.9 is considered excellent, 0.7–0.8 is considered acceptable (AC), 0.5–0.7 is considered poor in terms of accuracy of the test under consideration.

**Table 4:** Crude OR with 95% confidence intervals (CI) of clinical and demographic variables for higher ORM concentrations (dichotomized according to the identified optimal cut-off value), using univariate logistic regression.

ORM Concentrations (Categorized According to the Optimal Cut-off (≤1.20 g/L vs. >1.20 g/L)			
Variable	OR (95% CI)	*p*	C
Age	0.985 (0.949–1.022)	0.423	0.440
Gender			
Female	Reference		
Male	0.800 (0.311–2.056)	0.643	0.476
Smoking			
No	Reference		
Yes	0.595 (0.154–2.292)	0.450	0.472
WFNS	1.944 (1.378–2.741)	<0.001	0.738
mFisher-score	1.854 (1.095–3.139)	0.022	0.676
CRP	1.023 (1.005–1.042)	0.014	0.690
Neutrophile count	1.191 (1.040–1.363)	0.011	0.690

C = the area under an ROC curve (also known as c-statistic) provides an overall measure of diagnostic accuracy, with the value of one representing perfect accuracy. OR = odds ratios; ORM, orosomucoid; WFNS, World Federation of Neurological Societies score; CRP, C-reactive protein.

## Data Availability

The datasets used and or analyzed in the current study are available from the corresponding author upon reasonable request.
